# P110β in the ventromedial hypothalamus regulates glucose and energy metabolism

**DOI:** 10.1038/s12276-019-0249-8

**Published:** 2019-04-26

**Authors:** Teppei Fujikawa, Yun-Hee Choi, Dong Joo Yang, Dong Min Shin, Jose Donato, Daisuke Kohno, Charlotte E. Lee, Carol F. Elias, Syann Lee, Ki Woo Kim

**Affiliations:** 1 Division of Hypothalamic Research, Department of Internal Medicine, UT Southwestern Medical Center, Dallas, TX 75390 USA; 2 Department of Pharmacology, UT Southwestern Medical Center, Dallas, TX 75390 USA; 3 Department of Cellular and Integrative Physiology, Long School of Medicine, UT Health San Antonio, San Antonio, TX USA; 4 Department of Oral Biology, BK21 PLUS, Yonsei University College of Dentistry, Seoul, 03722 Korea; 5 Department of Physiology and Biophysics, Institute of Biomedical Sciences, University of São Paulo, São Paulo, SP 05508000 Brazil; 6 Metabolic Signal Research Center, Institute for Molecular and Cellular Regulation, Gunma University, Maebashi, 371-8512 Japan; 7 Department of Molecular and Integrative Physiology, University of Michigan, Ann Arbor, MI USA

**Keywords:** Obesity, Experimental models of disease, Neuroscience, Obesity

## Abstract

Phosphoinositide 3-kinase (PI3K) signaling in hypothalamic neurons integrates peripheral metabolic cues, including leptin and insulin, to coordinate systemic glucose and energy homeostasis. PI3K is composed of different subunits, each of which has several unique isoforms. However, the role of the PI3K subunits and isoforms in the ventromedial hypothalamus (VMH), a prominent site for the regulation of glucose and energy homeostasis, is unclear. Here we investigated the role of subunit p110β in steroidogenic factor-1 (SF-1) neurons of the VMH in the regulation of metabolism. Our data demonstrate that the deletion of p110β in SF-1 neurons disrupts glucose metabolism, rendering the mice insulin resistant. In addition, the deletion of p110β in SF-1 neurons leads to the whitening of brown adipose tissues and increased susceptibility to diet-induced obesity due to blunted energy expenditure. These results highlight a critical role for p110β in the regulation of glucose and energy homeostasis via VMH neurons.

## Metabolism: Enzymatic subunit essential to brain’s glucose responses

A particular subunit of a critical signaling enzyme is needed for neurons inside the brain’s hypothalamus to properly regulate energy metabolism. Ki Woo Kim from Yonsei University College of Dentistry, Seoul, South Korea, and colleagues explored the role that the PI3K enzyme plays in neurons of the ventromedial area toward the front of the hypothalamus, a region involved in regulating hunger and metabolism. Deleting a subunit of PI3K called p110β, which is needed for enzymatic function, made mice less responsive to insulin, the hormone that keeps blood sugar levels at healthy levels. As well as having abnormal glucose metabolism, the mice converted more brown fat, which burns energy, into white fat, which stores energy. They were also more susceptible to diet-induced obesity. The findings point toward p110β as a potential drug target for treating diabetes.

## Introduction

Obesity and obesity-related metabolic diseases are major public health burdens^1^. The central nervous system (CNS) governs whole-body metabolism by sensing and responding to fluctuating levels of circulating cues, such as nutrients and hormones. Unraveling the neuronal mechanisms by which the CNS regulates metabolism is a fundamental step in the treatment of metabolic disease and recent scientific efforts in this area have led to a new class of Food and Drug Administration-approved anti-obesity drugs^2^.

The hypothalamus is an important region for the regulation of metabolism^3^. In particular, the ventral medial nucleus of the hypothalamus (VMH) has been known since the early 1940s, to play a critical role in the regulation of glucose and energy balance^4,5^. However, the molecular blueprint underlying the VMH regulation of glucose and energy homeostasis remains unclear. Phosphoinositide 3-kinase (PI3K) is critical for the integration of metabolic hormone cues. It is composed of the regulatory subunit p85 and the catalytic subunit p110, and each subunit comprised several variant forms. Previously, we demonstrated that mice lacking p110α in the VMH are more prone to high-fat diet (HFD)-induced obesity and obesity-related metabolic disturbances^6^. Recent studies have shown distinct metabolic roles for each subunit/variant in proopiomelanocortin (POMC) and agouti-related peptide (AgRP) neurons of the arcuate nucleus (ARC) of the hypothalamus^7–9^. These studies indicate that, at least in ARC neurons, p110β plays a greater role in the regulation of metabolism than does p110α. Although electrophysiological approaches suggest that p110β is required for leptin and insulin action in the VMH^10^, the specific metabolic roles of each of the PI3K subunits in VMH neurons are not well understood. Here we investigated the role of p110β in the VMH in the regulation of glucose and energy metabolism.

## Materials and methods

### Animal care and generation of tissue-specific KO mice

All experimental procedures were approved by the Institutional Animal Care and Use Committees at UT Southwestern (Dallas, TX) and Yonsei University College of Medicine. Mice were kept at room temperature (22 °C–24 °C) with a 12 h light/dark cycle (lights on at 06:00 h) and fed a normal mouse chow diet (4% fat diet; 7001; Harlan Laboratories) or a HFD (Research Diet #D12331; 58% kcal from fat, 26% from sucrose, 5.56 kcal/g) with water provided ad libitum. To generate VMH-specific p110β knockout (KO) (p110β KO^sf1^) mice, males that were homozygous for the floxed (F) *p110β* allele^11^ and heterozygous for the *Sf-1*-Cre transgene^12^ were crossed with female mice homozygous for the floxed *p110β* allele. Littermate mice homozygous for the floxed *p110β* allele (*p110β*^*F/F*^) served as controls (Ctr). All experimental mice were on a mixed C57BL/6;129S6/SvEv background.

### Protein and mRNA analyses

All samples were collected between 1300 and 1500 h for quantitative PCR (Q-PCR) analysis. Total RNA was isolated using Trizol reagent (Invitrogen, Carlsbad, CA) and reverse transcribed with a SuperScript First-Strand Synthesis System (Invitrogen) for reverse transcriptase PCR (RT-PCR). Real-time PCR (Q-PCR) was performed using an ABI 7900 HT Sequence Detection System (Applied Biosystems, Foster City, CA). The Q-PCR primers used for the TaqMan method (Applied Biosystems) are as follows: 18S (ABI, Hs99999901), pik3ca (ABI, Mm00435673_m1), pik3cb (ABI, Mm00659576_m), pik3r1 (ABI, Mm00808818_s1), pik3c2a (ABI, Mm00478162_m1), β-adrenergic receptor 3 (β3-AR) (ABI, Mm02601819_g1), Cidea (ABI, Mm00432554_m1), PGC1α (ABI, Mm01208835_m1), PPARγ (ABI, Mm01184322_m1), PRDM16 (ABI, Mm01266507_g1), uncoupling protein 1 (UCP1) (ABI, Mm01244861), and UCP3 (ABI, Mm01163394_m1).

For protein analysis, tissues from control and p110β KO^sf1^ mice were homogenized in lysis buffer [20 mM Tris, 5 mM EDTA, and NP40 1% (v/v)] containing protease inhibitors (P2714 Sigma, St. Louis, MO, resolved by SDS-polyacrylamide gel electrophoresis and finally transferred to a nitrocellulose membrane. After blocking the membrane with 5% non-fat milk, proteins were detected using the following commercially available antisera: UCP1 (Abcam, Cambridge, MA, 1:5000), GAPDH (Santa Cruz Biotech, Santa Cruz, CA, 1:5000), phosphorylation of AKT (pAKT) (Cell Signaling Technology, 1:2000), and pFoxO1 (Cell Signaling Technology, 1:1000).

### In situ hybridization

RNA in situ hybridization was performed on every fourth serial section from the brains of control and p110β KO^sf1^ mice^13–17^ (*n* = 5 for each genotype). Before hybridization, brain sections were mounted onto SuperFrost Plus slides (Fisher Scientific) and stored at −20 °C. Before hybridization, sections were fixed in 4% formaldehyde for 20 min, dehydrated in ascending concentrations of ethanol, cleared in xylene for 15 min, rehydrated in descending concentrations of ethanol, and placed in prewarmed 0.01 M sodium citrate buffer pH 6.0. Sections were pretreated for 10 min in a microwave, dehydrated in ethanol, and air-dried. The p110β riboprobe was generated by in vitro transcription with ^35^S-UTP. The ^35^S-labeled probe was diluted (10^6^ dpm/mL) in hybridization solution containing 50% formamide, 10% dextran sulfate, and 1× Denhardt’s solution (Sigma). The hybridization solution (120 µl) was applied to each slide and incubated overnight at 56 °C. Sections were then treated with 0.002% RNAase A solution and submitted to stringency washes in decreasing concentrations of sodium chloride/sodium citrate buffer. Sections were dehydrated and enclosed in X-ray film cassettes with BMR-2 film (Kodak) for 72 h. Slides were dipped into an NTB2 autoradiographic emulsion (Kodak), dried, and stored at 4 °C for 25 days. Slides were developed with a D-19 developer (Kodak). The p110β probe was produced from PCR fragments amplified with ExTaq DNA polymerase (Takara) from cDNA generated with SuperScript II Reverse Transcriptase (Invitrogen) for RT-PCR from total mouse hypothalamic RNA. The p110β probe comprises positions 502–762 of the NCBI reference sequence NM_029094.3 and spans exon 4 of the *Pik3cb* gene. This region is flanked by LoxP sites and, therefore, this probe can be used to identify the Cre-mediated deletion of the *Pik3cb* gene. All images were captured with a Nikon E1000 automated microscope installed with a Nikon digital camera (DXM 1200F; Nikon, Melville, NY).

### Metabolic cage studies

A combined indirect calorimetry system (CaloSys Calorimetry System, TSE Systems, Inc., Bad Homburg, Germany) was used for all metabolic studies. Experimental animals were acclimated for 5 days in a home cage with food and water. The room temperature for all metabolic studies was maintained at 22 °C with a 12 h light/dark cycle. Heat generation, O_2_ consumption, and CO_2_ production were measured after acclimation, and the relationship between metabolic rate and body mass was normalized to metabolic body size (body weight 0.75) unless otherwise noted. During this time, ambulatory and rearing activities were also monitored with infrared beams.

To assess diet-induced thermogenesis, chow-fed mice with matched body weights were acclimatized in the TSE metabolic chambers as described above, followed by continuous monitoring of the metabolic rate. Chow was provided from day 1 to day 4 and replaced with a HFD at 17:00 h of day 4. Metabolic parameters were measured for 3 additional days. The ΔVO_2_ was calculated by the VO_2_ difference before and after the HFD.

### Hormone measurement

Corticosterone levels were measured as previously described^6^. Briefly, psychosocial stress was given to male mice by housing for 30 min in groups of four animals after 3 days of isolation. Trunk blood for corticosterone measurements was taken by decapitation at the indicated times (Supplementary Table 1). For follicle-stimulating hormone (FSH), luteinizing hormone (LH), testosterone, epinephrine, and norepinephrine measurements, serum and/or plasma were obtained between 14:00 and 15:30 h. The blood samples for corticosterone, FSH, LH, testosterone, epinephrine, and norepinephrine levels were sent for analysis to either the Ligand Assay & Analysis Core at the University of Virginia or the Hormone Assay & Analytical Services Core, Vanderbilt Diabetes Research and Training Center.

### VMH dissection for western blotting and Q-PCR analyses

To assess leptin-mediated AKT and forkhead box-containing protein of the O subfamily-1 (FoxO1) phosphorylation, body weight-matched 9- to 13-week-old male mice were fasted for 18 h and given murine leptin (5 mg/kg body weight, Sigma, St. Louis, MO) or pyrogen-free saline (Sigma, St. Louis, MO). After 40 min, the animals were transcardially perfused with 10% formalin. A coronal slice between bregma −1.22 mm and −2.06 mm was made, and then the VMH was microdissected with a scalpel under a microscope. All samples were immediately frozen on dry ice. Protein lysate was prepared from the VMH sample and used for western blotting analysis as described above.

To measure mRNA levels in the VMH of control and p110β KO^sf1^ male mice, mice were decapitated after deep anesthesia. The VMH was microdissected with a scalpel under a microscope as described above. All samples were immediately frozen on dry ice. Total mRNA was extracted and used for Q-PCR analyses.

### Histology

All tissues were fixed in 10% neutral buffered formalin and either transferred to 1× phosphate-buffered saline followed by paraffin embedding or cryoembedded after sucrose infiltration for hematoxylin and eosin (H&E), Nissl, pSTAT3, or Oil Red O staining.

### Body weight and composition

The body weight of control and p110β KO^sf1^ mice fed a normal chow diet (NCD) was monitored weekly from weaning (4 weeks old) to 21 weeks. The body composition of control and p110β KO^sf1^ mice was determined using a Bruker Minispec mq10 nuclear magnetic resonance analyzer (The Woodlands, TX).

### GTT and ITT

The glucose tolerance test (GTT) was performed as previously described^18^. Male p110β KO^sf1^ mice and control littermates between the ages of 20–23 weeks were fasted for 18 h with water provided ad libitum. After fasted glucose levels were measured, glucose was administered via intraperitoneal (i.p.) injection (1.5 g/kg body weight). Blood glucose levels were measured from blood sampled from tail nicks at 20, 40, 60, 90, and 120 min after injection. Blood glucose levels were determined by the glucose oxidase method using a commercial glucometer (Ascensia Contour; Bayer HealthCare, Mishawaka, IN). For the insulin tolerance test (ITT), male mice between the ages of 20–23 weeks were fasted for 2 h with water provided ad libitum. After measurements of basal glucose levels, insulin (0.8 U/kg, Eli Lilly and Company, HI-210, Indianapolis, IN) was administered via i.p. injection. Blood glucose levels were monitored as described above.

### Data analysis

The data are presented as the mean ± SEM, as indicated in each figure legend. Statistical significance was determined by Student’s *t*-test or two-way analysis of variance. GraphPad Prism, version 5.0a (GraphPad, San Diego, CA), was used for all statistical analyses and *P* < 0.05 was considered a statistically significant difference.

## Results

### Generation of SF-1 neuron (VMH)-specific p110β KO mice

**Fig. 1:**
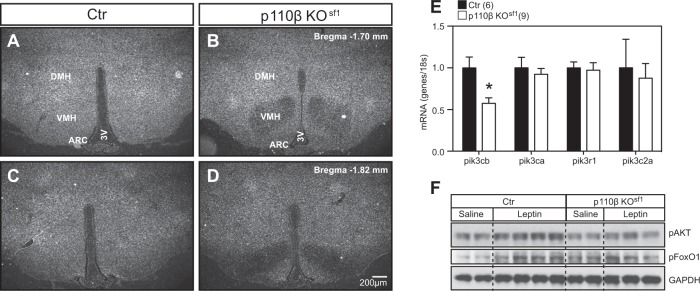
Deletion of p110β is restricted to SF-1 neurons of the VMH. Expression of p110β detected by RNA in situ hybridization in the anterior VMH of **a** control and **b** p110β KO^sf1^ mice, and the posterior VMH of **c** control and **d** p110β KO^sf1^ mice. **e** mRNA expression of *p110β*, *p110α*, *p85α*, and *PI3K-C2α* in the VMH of control and p110β KO^sf1^ mice. **f** The VMH was collected after i.p. administration of leptin (5 mg/kg body weight) and the levels of pAKT and pFoxO1 were measured in control and p110β KO^sf1^ mice. The number of mice in each group is indicated in the figure. *P < 0.05 by Student’s *t*-test. Data are shown as mean ± SEM. Scale bar = 200 μm. Abbreviations: 3V, third ventricle; ARC, arcuate nucleus; Ctr, control; DMH, dorsomedial nucleus of the hypothalamus

p110β is ubiquitously expressed and mice lacking p110β in the VMH were generated by crossing floxed *p110β* mice^11^ with steroidogenic factor-1 (*Sf-1*) Cre mice (p110β KO^sf1^)^12^, which in the CNS, express Cre recombinase exclusively in the VMH. Histological analyses confirmed that the deletion of p110β was confined to the VMH (Fig. 1a–d) without disturbing VMH cytoarchitecture (Supplementary Fig. 1). Q-PCR analysis of RNA isolated from the VMH showed that p110β was significantly reduced, and that the expression of the remaining isoforms and subunits was unchanged (Fig. 1e). Peripherally, SF-1 is also expressed in the pituitary, adrenal glands, and gonads, which are important tissues for the regulation of metabolism. We therefore examined these tissues for morphological changes and measured the circulating levels of corticosterone (normal and stressed), testosterone, FSH, and LH. We found similar tissue morphology and hormone parameters between the two genotypes, indicating that the hypothalamic–pituitary–adrenal and hypothalamic–pituitary–gonadal axes were intact (Supplementary Fig. 2 and Supplementary Table 1). These data suggest that the metabolic phenotype of the p110β KO^sf1^ mice described in this study is not secondary to disruptions in these hormones.

To determine whether the deletion of p110β in SF-1 neurons altered PI3K signaling in the VMH^19^, we measured the pAKT and FoxO1 in the VMH after intraperitoneal leptin administration (5 mg/kg). Although leptin administration activated pAKT and pFoxO1 in the VMH of control mice, this effect was significantly blunted in mice that lacked p110β (Fig. 1f). In contrast, the activation of pSTAT3 by leptin was comparable (Supplementary Fig. 3). These results indicate that p110β in SF-1 neurons of the VMH is necessary for the normal activation of the PI3K pathway.

### p110β isoform in the VMH is required for normal glucose homeostasis

**Fig. 2:**
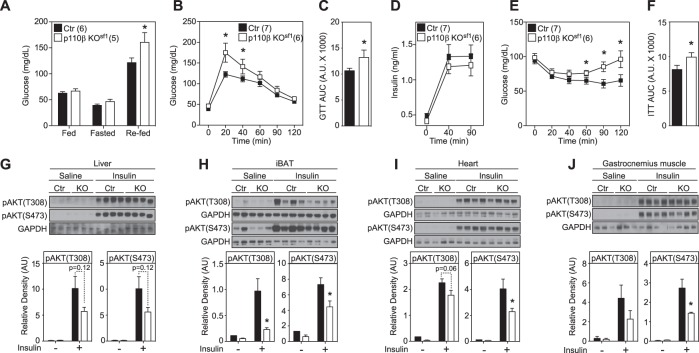
Deletion of p110β in SF-1 neurons disrupts glucose and insulin homeostasis. **a** Glucose levels in fed (3 h fasted), fasted (24 h), and refed (1 h) male mice. **b** GTT [significant interaction (two-way repeated ANOVA, F_5.62_ = 37.65, *p* < 0.0001)]. **c** Area under the curve (AUC) from **b**. **d** Insulin levels during the GTT. **e** ITT [significant interaction (two-way repeated ANOVA, F_1.66_ = 14.84, *p* = 0.00003)]. **f** Area under the curve (AUC) from **e**. Blunted insulin-induced pAKT activation in the **g** liver, **h** iBAT, **i** heart, and **j** gastrocnemius muscle of p110β KO^sf1^ mice. Number of mice in each group is indicated in the legends or directly in the figures. Data are shown as the mean ± SEM. *P < 0.05 by Student’s *t*-test

To investigate the role of p110β in the regulation of energy homeostasis, we first examined several metabolic parameters in mice fed a NCD. No differences were observed in body weight, body composition, leptin and insulin levels, food intake, oxygen consumption (VO_2_), locomotor activity, or respiratory exchange ratio (RER) between littermate controls and p110β KO^sf1^ mice (Supplementary Fig. 4). Numerous studies suggest that the VMH is a key brain site for the regulation of glucose homeostasis through the modulation of the autonomic nervous system^20–23^. For instance, microinjection of leptin or orexin into the VMH increases glucose uptake and enhances insulin sensitivity, and VMH-mediated glucose uptake is blocked by inhibition of the sympathetic nervous system (SNS)^24–26^. Although we found no significant differences in the glucose levels of mice fed a NCD, glucose levels during the refeeding period following a 24 h fast were significantly elevated in p110β KO^sf1^ mice compared with control mice (Fig. 2a). Furthermore, p110β KO^sf1^ mice exhibited blunted glucose and insulin sensitivity in response to both i.p. GTTs and ITTs (Fig. 2b–f). Notably, previous studies have shown that the deletion of p110α in the VMH does not affect glucose metabolism in NCD-fed mice^6^. Our data suggest that glucose homeostasis by SF-1 neurons in the VMH is uniquely mediated by the p110β subunit.

Serum insulin levels obtained during the course of the GTT were unaltered (Fig. 2d), suggesting an impairment in insulin sensitivity rather than impaired insulin secretion from pancreatic β-cells. Therefore, we measured insulin sensitivity in peripheral tissues, including the liver, interscapular brown adipose tissue (iBAT), heart, and muscle, by monitoring the activation of pAKT after i.p. injection of insulin^27^. The insulin-mediated activation of pAKT was decreased in p110β KO^sf1^ mice in all tissues examined, including the iBAT, heart, and muscle, compared with control littermates (Fig. 2g–j). These results strongly suggest that the blunted insulin sensitivity in these peripheral tissues contributes to altered whole-body glucose homeostasis in p110β KO^sf1^ mice.

### Increased whitening of iBAT and decreased energy expenditure in p110β KO^sf1^ mice

**Fig. 3:**
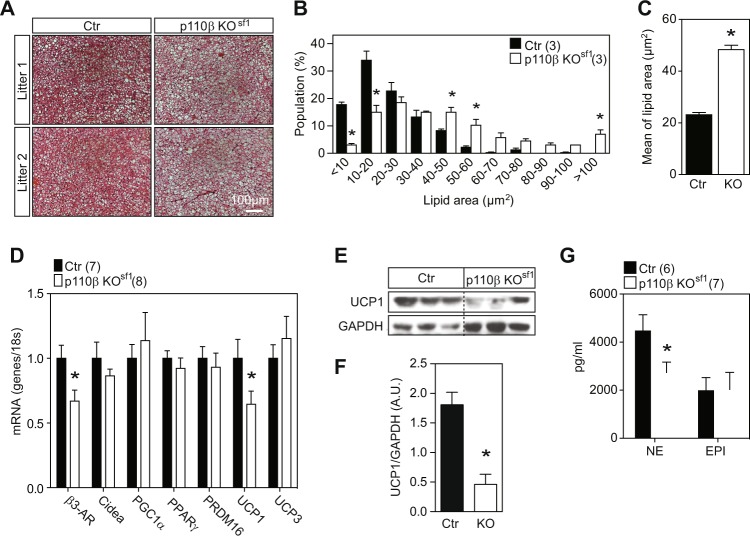
Deletion of p110β in SF-1 neurons leads to the whitening of iBAT. **a** Hematoxylin and eosin staining of iBAT from control and p110β KO^sf1^ mice. **b** Distribution (% of population) of lipid droplets from **a** [significant interaction (two-way ANOVA, F_1.20_ = 18.02, *p* = 0.0001)]. **c** Mean lipid area (μm^2^) of the droplets from **a**. **d** mRNA levels of genes in iBAT regulating BAT programming. **e** UCP1 protein levels in the iBAT of control and p110β KO^sf1^ mice. **f** Average UCP1 protein levels in the iBAT of control and p110β KO^sf1^ mice. **g** Plasma norepinephrine and epinephrine levels in chow-fed males. Number of mice in each group is indicated in the legends or directly in the figures. Data are shown as the mean ± SEM. *P < 0.05 by Student’s *t*-test. EPI, epinephrine; Ctr, control; NE, norepinephrine

The VMH is a critical brain site mediating sympathetic tone to the iBAT^25,28,29^. A disruption in β-adrenergic signaling causes iBAT lipid accumulation^30,31^, a process known as “whitening”^31^. Notably, the activation of pAKT in the iBAT after insulin administration was significantly blunted in p110β KO^sf1^ mice (Fig. 2h). H&E staining revealed an increase in lipid droplets in p110β KO^sf1^ mice (Fig. 3a–c). In addition, the RNA levels of β3-AR and UCP1, and the protein levels of UCP1 were significantly reduced in the iBAT of p110β KO^sf1^ mice (Fig. 3d–f). Moreover, plasma norepinephrine, a neurotransmitter released by sympathetic nerve terminals, was decreased in p110β KO^sf1^ mice (Fig. 3g). Our study demonstrates that the deletion of p110β in SF-1 neurons hampers sympathetic activity and leads to the whitening of iBAT. Collectively, these data suggest that p110β expression in the VMH is a key module to maintain BAT programming.

**Fig. 4:**
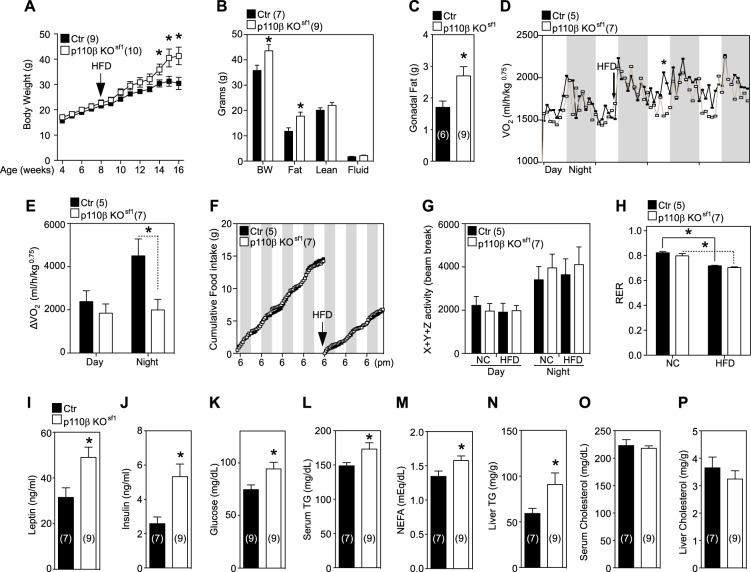
Diet-induced obesity and blunted energy expenditure in p110β KO^sf1^ mice. **a** Body weights of male mice fed a HFD for 8 weeks. **b** Body composition (18–20 weeks) of male mice fed a HFD for 8 weeks. **c** Gonadal fat pad weights of male mice fed a HFD for 8 weeks. **d** Changes in O_2_ consumption before and after HFD feeding. The star indicates a significant difference at a specific time point (15:00 h). **e** Temporal change in O_2_ consumption before and after HFD feeding. **f** Cumulative food intake before and after HFD feeding. **g** Total movement. **h** Respiratory exchange ratio. **i** Leptin, **j** insulin, **k** fasted glucose, **l** serum triglyceride (TG), **m** serum free fatty acid, **n** liver TG, **o** serum cholesterol, and **p** liver cholesterol levels in control and p110β KO^sf1^ mice fed a HFD. Number of animals examined is expressed in parentheses in each graph. Data are shown as the mean ± SEM. *P < 0.05 by Student’s *t*-test

As p110β KO^sf1^ mice displayed changes in sympathetic tone, we postulated that metabolic stress would alter metabolic homeostasis in p110β KO^sf1^ mice. Of note, a HFD decreases UCP1, PGC1α, and other genes, which are important for maintaining BAT programming^31^. To address this hypothesis, metabolic stress was induced by challenging mice with a HFD and assessing the metabolic response of p110β KO^sf1^ mice. The body weight of p110β KO^sf1^ mice began to diverge from that of control mice after 6 weeks of HFD feeding (Fig. 4a). The increased body weight was caused by increased fat mass but not lean mass (Fig. 4b, c). Indirect calorimetry studies revealed significantly decreased oxygen consumption in p110β KO^sf1^ mice, without changes in food intake, movement, or the RER during HFD feeding (Fig. 4d–h). These data imply that PI3K activity in SF-1 neurons of the VMH might be necessary for the regulation of energy expenditure, especially under high-calorie conditions. Serum analysis showed elevated levels of leptin, insulin, fasted glucose, triglyceride (TG), and free fatty acid in HFD-fed p110β KO^sf1^ mice (Fig. 4i–m). In addition, HFD-fed p110β KO^sf1^ mice exhibited increased liver TG (Fig. 4n) but not serum or liver cholesterol (Fig. 4o, p). These results indicate that the p110β subunit in the VMH might be involved in the regulation of metabolic homeostasis.

## Discussion

Although the metabolic importance of PI3K has been shown in several tissues, little is known about its function in the hypothalamus^6,9,11^. In this study, we specifically deleted the p110β isoform of PI3K from SF-1 neurons of the VMH. We found that p110β in the VMH, possibly through actions on the autonomic nervous system, is required for energy homeostasis and the maintenance of normal glucose and insulin sensitivity. p110α and p110β are class IA PI3K isoforms, and studies using global KO mice have suggested that each isoform has distinct metabolic functions^32,33^. Notably, the deletion of class I PI3K isoforms in ARC POMC or AgRP neurons revealed that p110β has a greater contribution than does p110α to metabolic parameters, such as body weight, food intake, and leptin-mediated neuronal excitability^9,34^. Our studies have extended these findings to the VMH. We previously showed that p110α deletions in the VMH affect diet-induced obesity but not the basal metabolic rate^6^. Our current study shows that p110β in SF-1 neurons of the VMH plays a much broader role, affecting glucose and insulin homeostasis and BAT function.

The VMH is well known to regulate many physiological processes, including energy expenditure, reproduction, defensive behavior, food intake, carbohydrate and fat metabolism, and metabolic adaptation^6,12,18,23,29,35–57^. In 1966, Shimazu et al.^35^ demonstrated that electric stimulation of the VMH remarkably increased blood glucose and suggested the important role of the VMH in the regulation of glucose metabolism^56,57^. Previous reports have indicated that microinjection of leptin into the VMH can stimulate glucose uptake into the peripheral tissues, including skeletal muscle^25^. We recently found that the p110β subunit is required for leptin-induced depolarization in SF-1 neurons of the VMH^10^. Collectively, these studies suggest that the deletion of p110β in SF-1 neurons may compromise leptin’s glucoregulatory actions, leading to refractory responses to the GTT. Interestingly, we found that p110β KO^sf1^ mice exhibited glucose intolerance under refed conditions, with no significant body weight change, and exhibited diet-induced obesity, with significantly increased fasted glucose levels. p110β KO^sf1^ mice exhibited insulin insensitivity in the iBAT, heart, and gastrocnemius muscle. These results highly imply that the higher glucose level in p110β KO^sf1^ mice might be the result of decreased glucose uptake and insulin sensitivity mediated by decreased sympathetic tone.

A recent paper showed that SNS input is necessary for maintaining the thermogenic properties of BAT^31^. Disruption of the SNS signaling pathway leads to a whitening of BAT accompanied by a reduction in mitochondrial activity and the accumulation of lipid droplets^51^. In fact, *ob/ob*^58^ and DIO^31^ mice show impaired SNS and BAT whitening. The VMH regulates BAT function via the SNS^24,25,36,41^. Lesions in the VMH have been shown to cause mitochondrial dysfunction and to reduce fatty acid oxidation^59–61^, indicating that an intact VMH is important for maintaining BAT function. Lower levels of norepinephrine together with increased iBAT whitening in p110β KO^sf1^ mice suggest that p110β in the VMH might be a critical component for the SNS-mediated BAT pathway, while further analyses including the direct visualization of sympathetic nerve fibers are necessary.

Our study supports the notion that the VMH plays a critical role in regulating metabolic adaptations under conditions requiring high-energy expenditure, such as a HFD and exercise^6,12,48,50,53,55,62^. The regulation of energy expenditure by the VMH is known to be mediated by the SNS; however, the precise neuronal pathway linking the SNS and the VMH has not yet been precisely determined. Genetic tracing experiments revealed that SF-1 neurons project to several brain nuclei that regulate SNS function^63^; thus, future studies using emerging techniques such as channel rhodopsin-assisted neurocircuit mapping^64^ may provide further insights into the functional pathways linking the SNS and the VMH. In summary, the current study suggests that pharmaceutical therapies that target PI3K in a tissue- and isoform-specific manner may prove beneficial toward ameliorating metabolic syndrome, especially diabetes.

## Supplementary information

Supplementary Figure Legends

Supplementary Figure 1

Supplementary Figure 2

Supplementary Figure 3

Supplementary Figure 4

Supplementary Table 1

## References

[1] Dobbs, R. et al. How the world could better fight obesity (The McKinsey Global Institute, 2014).

[2] Gautron L, Elmquist JK, Williams KW (2015). Neural control of energy balance: translating circuits to therapies. Cell.

[3] Morton GJ, Meek TH, Schwartz MW (2014). Neurobiology of food intake in health and disease. Nat. Rev. Neurosci..

[4] Hetherington AW (1941). The relation of various hypothalamic lesions to adiposity and other phenomena in the rat. Am. J. Physiol..

[5] Choi YH, Fujikawa T, Lee J, Reuter A, Kim KW (2013). Revisiting the ventral medial nucleus of the hypothalamus: the roles of SF-1 neurons in energy homeostasis. Front. Neurosci..

[6] Xu Y (2010). PI3K signaling in the ventromedial hypothalamic nucleus is required for normal energy homeostasis. Cell Metab..

[7] Hill JW (2009). Phosphatidyl inositol 3-kinase signaling in hypothalamic proopiomelanocortin neurons contributes to the regulation of glucose homeostasis. Endocrinology.

[8] Hill JW (2010). Direct insulin and leptin action on pro-opiomelanocortin neurons is required for normal glucose homeostasis and fertility. Cell Metab..

[9] Al-Qassab H (2009). Dominant role of the p110beta isoform of PI3K over p110alpha in energy homeostasis regulation by POMC and AgRP neurons. Cell Metab..

[10] Sohn JW (2016). Leptin and insulin engage specific PI3K subunits in hypothalamic SF1 neurons. Mol. Metab..

[11] Jia S (2008). Essential roles of PI(3)K-p110beta in cell growth, metabolism and tumorigenesis. Nature.

[12] Dhillon H (2006). Leptin directly activates SF1 neurons in the VMH, and this action by leptin is required for normal body-weight homeostasis. Neuron.

[13] Kishi T (2003). Expression of melanocortin 4 receptor mRNA in the central nervous system of the rat. J. Comp. Neurol..

[14] Kim KW (2008). Steroidogenic factor 1 regulates expression of the cannabinoid receptor 1 in the ventromedial hypothalamic nucleus. Mol. Endocrinol..

[15] Zhao L (2008). Central nervous system-specific knockout of steroidogenic factor 1 results in increased anxiety-like behavior. Mol. Endocrinol..

[16] Tong Q (2007). Synaptic glutamate release by ventromedial hypothalamic neurons is part of the neurocircuitry that prevents hypoglycemia. Cell Metab..

[17] Elias CF (2000). Chemical characterization of leptin-activated neurons in the rat brain. J. Comp. Neurol..

[18] Bingham NC, Anderson KK, Reuter AL, Stallings NR, Parker KL (2008). Selective loss of leptin receptors in the ventromedial hypothalamic nucleus results in increased adiposity and a metabolic syndrome. Endocrinology.

[19] Williams KW, Scott MM, Elmquist JK (2011). Modulation of the central melanocortin system by leptin, insulin, and serotonin: co-ordinated actions in a dispersed neuronal network. Eur. J. Pharm..

[20] Cotero VE, Routh VH (2009). Insulin blunts the response of glucose-excited neurons in the ventrolateral-ventromedial hypothalamic nucleus to decreased glucose. Am. J. Physiol. Endocrinol. Metab..

[21] Borg MA, Sherwin RS, Borg WP, Tamborlane WV, Shulman GI (1997). Local ventromedial hypothalamus glucose perfusion blocks counterregulation during systemic hypoglycemia in awake rats. J. Clin. Invest..

[22] Borg WP (1994). Ventromedial hypothalamic lesions in rats suppress counterregulatory responses to hypoglycemia. J. Clin. Invest..

[23] Borg WP, Sherwin RS, During MJ, Borg MA, Shulman GI (1995). Local ventromedial hypothalamus glucopenia triggers counterregulatory hormone release. Diabetes.

[24] Haque MS (1999). Role of the sympathetic nervous system and insulin in enhancing glucose uptake in peripheral tissues after intrahypothalamic injection of leptin in rats. Diabetes.

[25] Minokoshi Y, Haque MS, Shimazu T (1999). Microinjection of leptin into the ventromedial hypothalamus increases glucose uptake in peripheral tissues in rats. Diabetes.

[26] Shiuchi T (2009). Hypothalamic orexin stimulates feeding-associated glucose utilization in skeletal muscle via sympathetic nervous system. Cell Metab..

[27] Lu M (2012). Insulin regulates liver metabolism in vivo in the absence of hepatic Akt and Foxo1. Nat. Med..

[28] Rothwell NJ, Stock MJ (1979). A role for brown adipose tissue in diet-induced thermogenesis. Nature.

[29] Minokoshi Y, Saito M, Shimazu T (1986). Sympathetic denervation impairs responses of brown adipose tissue to VMH stimulation. Am. J. Physiol..

[30] Bachman ES (2002). betaAR signaling required for diet-induced thermogenesis and obesity resistance. Science.

[31] Shimizu I (2014). Vascular rarefaction mediates whitening of brown fat in obesity. J. Clin. Invest..

[32] Foukas LC (2006). Critical role for the p110alpha phosphoinositide-3-OH kinase in growth and metabolic regulation. Nature.

[33] Ciraolo E (2008). Phosphoinositide 3-kinase p110beta activity: key role in metabolism and mammary gland cancer but not development. Sci. Signal..

[34] Hill JW (2008). Acute effects of leptin require PI3K signaling in hypothalamic proopiomelanocortin neurons in mice. J. Clin. Invest..

[35] Shimazu T, Fukuda A, Ban T (1966). Reciprocal influences of the ventromedial and lateral hypothalamic nuclei on blood glucose level and liver glycogen content. Nature.

[36] Perkins MN, Rothwell NJ, Stock MJ, Stone TW (1981). Activation of brown adipose tissue thermogenesis by the ventromedial hypothalamus. Nature.

[37] Shimazu T, Ishikawa K (1981). Modulation by the hypothalamus of glucagon and insulin secretion in rabbits: studies with electrical and chemical stimulations. Endocrinology.

[38] Takahashi A, Shimazu T (1981). Hypothalamic regulation of lipid metabolism in the rat: effect of hypothalamic stimulation on lipolysis. J. Auton. Nerv. Syst..

[39] Vander Tuig JG, Knehans AW, Romsos DR (1982). Reduced sympathetic nervous system activity in rats with ventromedial hypothalamic lesions. Life Sci..

[40] Sakaguchi T, Bray GA (1987). The effect of intrahypothalamic injections of glucose on sympathetic efferent firing rate. Brain Res. Bull..

[41] Sakaguchi T, Bray GA (1987). Intrahypothalamic injection of insulin decreases firing rate of sympathetic nerves. Proc. Natl Acad. Sci. USA.

[42] Sakaguchi T, Arase K, Bray GA (1988). Sympathetic activity and food intake of rats with ventromedial hypothalamic lesions. Int J. Obes..

[43] Vissing J, Wallace JL, Scheurink AJ, Galbo H, Steffens AB (1989). Ventromedial hypothalamic regulation of hormonal and metabolic responses to exercise. Am. J. Physiol..

[44] Sakaguchi T, Bray GA (1990). Ventromedial hypothalamic lesions attenuate responses of sympathetic nerves to carotid arterial infusions of glucose and insulin. Int J. Obes..

[45] Shimazu T, Sudo M, Minokoshi Y, Takahashi A (1991). Role of the hypothalamus in insulin-independent glucose uptake in peripheral tissues. Brain Res. Bull..

[46] Sudo M, Minokoshi Y, Shimazu T (1991). Ventromedial hypothalamic stimulation enhances peripheral glucose uptake in anesthetized rats. Am. J. Physiol..

[47] Musatov S (2007). Silencing of estrogen receptor alpha in the ventromedial nucleus of hypothalamus leads to metabolic syndrome. Proc. Natl Acad. Sci. USA.

[48] Klockener T (2011). High-fat feeding promotes obesity via insulin receptor/PI3K-dependent inhibition of SF-1 VMH neurons. Nat. Neurosci..

[49] Lin D (2011). Functional identification of an aggression locus in the mouse hypothalamus. Nature.

[50] Kim KW (2012). FOXO1 in the ventromedial hypothalamus regulates energy balance. J. Clin. Invest..

[51] Mobbs CV, Moreno CL, Poplawski M (2013). Metabolic mystery: aging, obesity, diabetes, and the ventromedial hypothalamus. Trends Endocrinol. Metab..

[52] Toda C (2013). Extracellular signal-regulated kinase in the ventromedial hypothalamus mediates leptin-induced glucose uptake in red-type skeletal muscle. Diabetes.

[53] Correa SM (2015). An estrogen-responsive module in the ventromedial hypothalamus selectively drives sex-specific activity in females. Cell Rep..

[54] Wang L, Chen IZ, Lin D (2015). Collateral pathways from the ventromedial hypothalamus mediate defensive behaviors. Neuron.

[55] Fujikawa, T. et al. SF-1 expression in the hypothalamus is required for beneficial metabolic effects of exercise. *Elife* **5**, pii: e18206 (2016). 10.7554/eLife.18206 PMC5119890 27874828

[56] Meek TH (2016). Functional identification of a neurocircuit regulating blood glucose. Proc. Natl Acad. Sci. USA.

[57] Stanley SA (2016). Bidirectional electromagnetic control of the hypothalamus regulates feeding and metabolism. Nature.

[58] Knehans AW, Romsos DR (1983). Norepinephrine turnover in obese (ob/ob) mice: effects of age, fasting, and acute cold. Am. J. Physiol..

[59] Seydoux J, Rohner-Jeanrenaud F, Assimacopoulos-Jeannet F, Jeanrenaud B, Girardier L (1981). Functional disconnection of brown adipose tissue in hypothalamic obesity in rats. Pflug. Arch..

[60] Saito M, Shimazu T (1984). Decreased rate of fatty acid synthesis in brown adipose tissue of hypothalamic obese rats. FEBS Lett..

[61] Seydoux J (1982). Decreased guanine nucleotide binding and reduced equivalent production by brown adipose tissue in hypothalamic obesity. Recovery after cold acclimation. FEBS Lett..

[62] Choi YH, Fujikawa T, Lee J, Reuter A, Kim KW (2013). Revisiting the ventral medial nucleus of the hypothalamus: the roles of SF-1 neurons in energy homeostasis. Front. Neurosci..

[63] Cheung CC, Kurrasch DM, Liang JK, Ingraham HA (2012). Genetic labeling of SF-1 neurons in mice reveals VMH circuitry beginning at neurogenesis and development of a separate non-SF-1 neuronal cluster in the ventrolateral VMH. J. Comp. Neurol..

[64] Sternson SM, Atasoy D, Betley JN, Henry FE, Xu S (2016). An emerging technology framework for the neurobiology of appetite. Cell Metab..

