# Supplementary material for: P110β in the ventromedial hypothalamus regulates glucose and energy metabolism
Source: Exp Mol Med. 2019 Apr 26;51(4):52. doi: 10.1038/s12276-019-0249-8 (PMC6486607; doi:10.1038/s12276-019-0249-8)
Supplement: Supplementary file 1 — Supplementary Figure Legends [file 12276_2019_249_MOESM1_ESM.docx]

**SUPPLEMENTARY FIGURE LEGENDS**

**Supplementary Figure 1. Nissl staining in (A) control and (B) p110β KO^sf1^ mice.**

**Supplementary Figure 2. Histology of the pituitary gland, adrenal gland, and testis in control and p110β KO^sf1^ mice.**

Sections from the (A and B) pituitary gland, (C and D) adrenal gland, and (E and F) testis from control and p110β KO^sf1^ mice were analyzed by H&E staining. Scale bar = 200 micrometers.

**Supplementary Figure 3. Activation of pSTAT3 in (A and C) control and (B and D) p110β KO^sf1^ mice after intraperitoneal leptin (5 mg/kg, 1 hr) injection.**

**Supplementary Figure 4. Metabolic phenotype of p110β KO^sf1^ mice fed a NCD.**

(A) Body weights of male mice fed a NCD.

(B) Body composition of male mice fed a NCD between the ages of 13-16 weeks.

(C) Leptin levels in male mice fed a NCD.

(D) Insulin levels in male mice fed a NCD.

(E) Cumulative food intake, (F) O_2_ consumption, (G) total movement, (H) RER and (I) glucose levels were measured. Numbers of animals examined are expressed in parentheses in each graph. Data are shown as the mean±S.E.M. BB, beam break counts.
