# Supplementary figures and images for: P110β in the ventromedial hypothalamus regulates glucose and energy metabolism
Source: Exp Mol Med. 2019 Apr 26;51(4):52. doi: 10.1038/s12276-019-0249-8 (PMC6486607; doi:10.1038/s12276-019-0249-8)

Supplementary Fig. 1

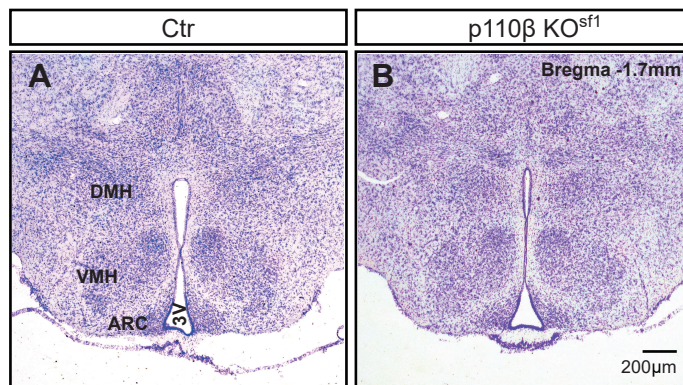

Supplement: Supplementary file 2 — Supplementary Figure 1 [file 12276_2019_249_MOESM2_ESM.pdf]

Supplementary Fig. 2

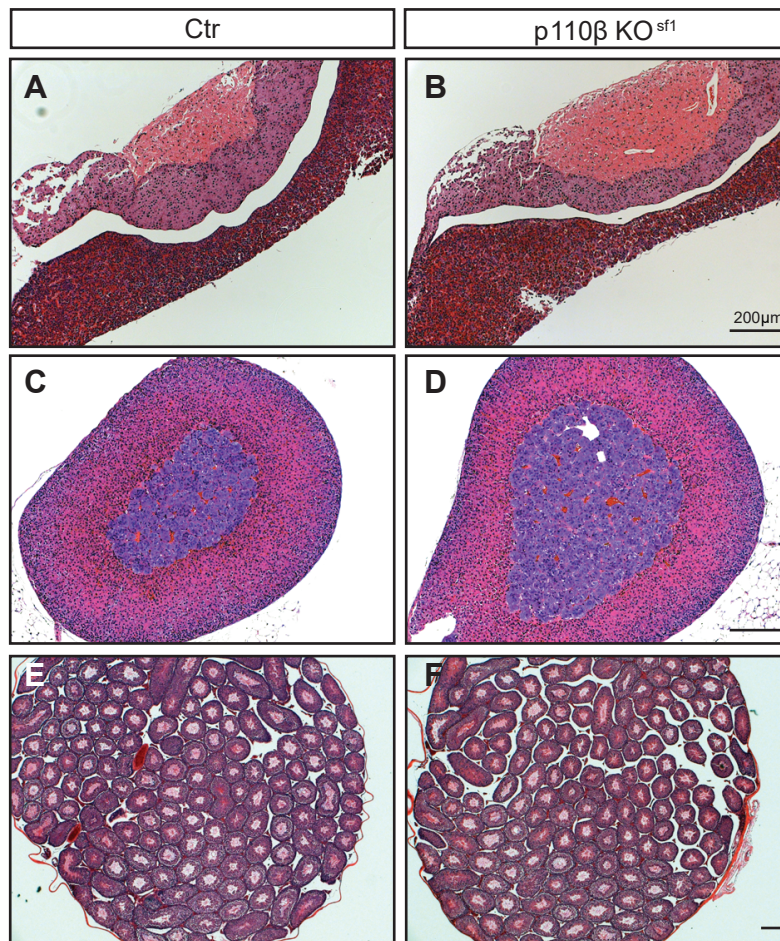

Supplement: Supplementary file 3 — Supplementary Figure 2 [file 12276_2019_249_MOESM3_ESM.pdf]

Supplementary Fig. 3

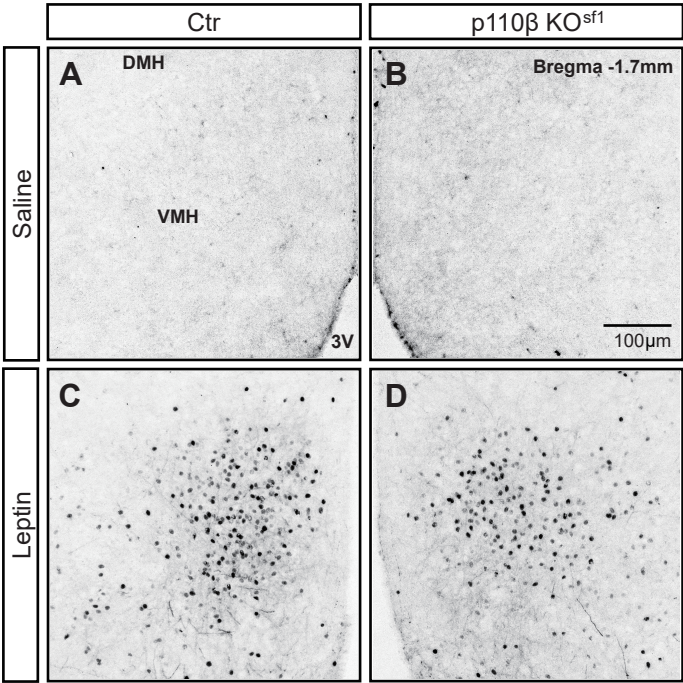

Supplement: Supplementary file 4 — Supplementary Figure 3 [file 12276_2019_249_MOESM4_ESM.pdf]

Supplementary Fig. 4

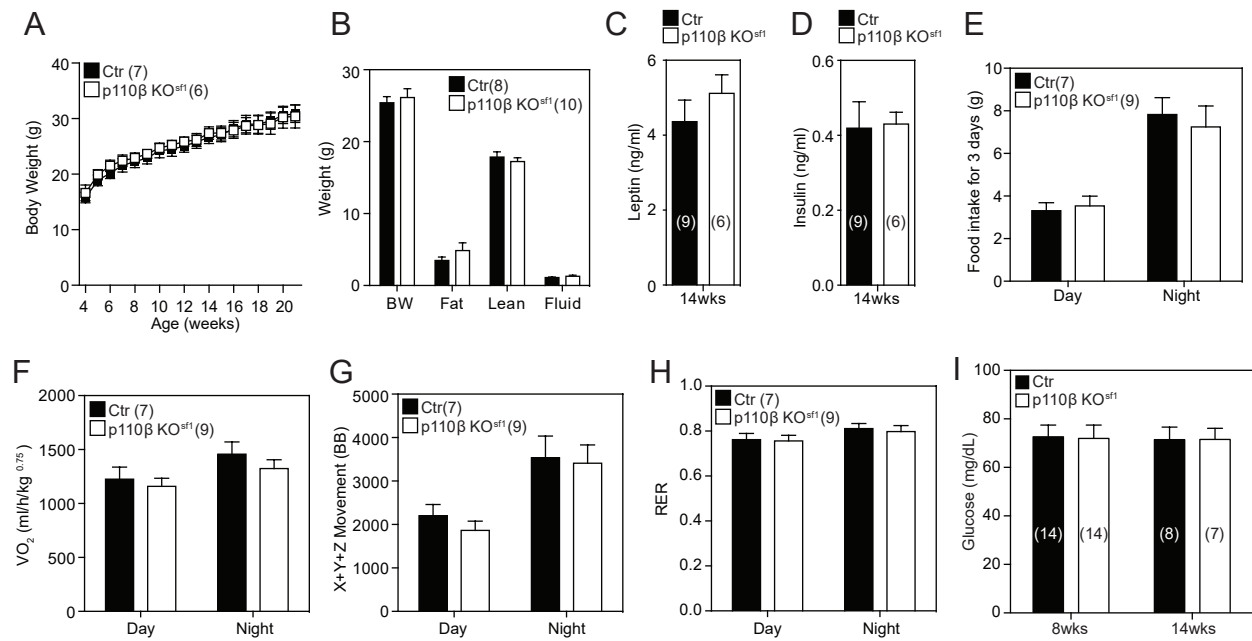

Supplement: Supplementary file 5 — Supplementary Figure 4 [file 12276_2019_249_MOESM5_ESM.pdf]
