# Supplementary material for: P110β in the ventromedial hypothalamus regulates glucose and energy metabolism
Source: Exp Mol Med. 2019 Apr 26;51(4):52. doi: 10.1038/s12276-019-0249-8 (PMC6486607; doi:10.1038/s12276-019-0249-8)
Supplement: Supplementary file 6 — Supplementary Table 1 [file 12276_2019_249_MOESM6_ESM.pdf]

Supplementary Table 1

Effect of p110β in the VMH on biochemical parameters

Parameter	WT	p110β KO^{sf1}	Age (wks)	Bleeding Time
Basal Corticosterone (ng/ml)-male	12.06±1.47 (7)	13.12±2.19 (7)	8-10	10:00-10:20
Basal Corticosterone (ng/ml)-male	24.04±1.61 (7)	22.16±1.98 (6)	22-24	14:00-15:30
Basal Corticosterone (ng/ml)-female	28.56±3.27 (10)	31.14±3.53 (9)	24	14:00-15:30
Stressed Corticosterone (ng/ml)-male	46.38±2.3 (7)	43.80±2.05 (7)	8-10	10:30-10:50
Testosterone (ng/ml)	0.35±0.17 (6)	0.21±0.08 (6)	23-31	14:00-15:30
FSH (ng/ml)	73.52±15.03 (6)	98.11±15.61 (6)	23-31	14:00-15:30
LH (ng/ml)	<0.24 (6)	<0.24 (6)	23-31	14:00-15:30

The data were expressed as mean±SEM and numbers of experiemental animals were indicated in parenthesis.
No statistical significance was detected between genotypes.
